# Significant reduction in manual annotation costs in ultrasound medical image database construction through step by step artificial intelligence pre-annotation

**DOI:** 10.1371/journal.pdig.0000738

**Published:** 2025-06-30

**Authors:** Fu Zheng, Liu XingMing, Xu JuYing, Tao MengYing, Yang BaoJian, Shan Yan, Ye KeWei, Lu ZhiKai, Huang Cheng, Qi KeLan, Chen XiHao, Du WenFei, He Ping, Wang RunYu, Ying Ying, Bu XiaoHui

**Affiliations:** 1 The Ultrasound Diagnosis Department, The 906th Hospital of Joint Logistics Support Force of PLA, Ningbo City, Zhejiang Province, China; 2 State Power Investment Corporation, Ningbo City, Zhejiang Province, China; 3 Third Rehabilitation Zone, Hangzhou Rehabilitation Center for Special Duty Service Men of the PLA Air Force, Hangzhou City, Zhejiang Province, China; 4 The Ultrasound Diagnosis Department, Hangzhou Special Service Sanatorium of the PLA Air Force, Hangzhou City, Zhejiang Province, China; Fundación Progreso y Salud: Junta de Andalucia Consejeria de Salud y Familias Fundacion Progreso y Salud, SPAIN

## Abstract

This study investigates the feasibility of reducing manual image annotation costs in medical image database construction by utilizing a step by step approach where the Artificial Intelligence model (AI model) trained on a previous batch of data automatically pre-annotates the next batch of image data, taking ultrasound image of thyroid nodule annotation as an example. The study used YOLOv8 as the AI model. During the AI model training, in addition to conventional image augmentation techniques, augmentation methods specifically tailored for ultrasound images were employed to balance the quantity differences between thyroid nodule classes and enhance model training effectiveness. The study found that training the model with augmented data significantly outperformed training with raw images data. When the number of original images number was only 1,360, with 7 thyroid nodule classifications, pre-annotation using the AI model trained on augmented data could save at least 30% of the manual annotation workload for junior physicians. When the scale of original images number reached 6,800, the classification accuracy of the AI model trained on augmented data was very close with that of junior physicians, eliminating the need for manual preliminary annotation.

## Author summary

Building high-quality medical image databases for training artificial intelligence (AI) applications often requires labor-intensive manual annotation by clinicians, which is also the case in ultrasound imaging. Additionally, class imbalances (e.g., rare vs. common nodule types) further exacerbate the complexity of this process.This study addresses these challenges by introducing a stepwise AI pre-annotation strategy combined with ultrasound-specific data augmentation to streamline thyroid nodule database construction. Using the YOLOv8 model, we developed a workflow where AI iteratively pre-annotates new image batches after training on prior data. Crucially, we augmented ultrasound images with simulated artifacts like defocus, acoustic shadows, and sidelobe effects, which are common in clinical practice but often overlooked in traditional augmentation, to improve model robustness and balance class representation. Our results show that this approach reduces junior physicians’ manual annotation workload by over 30% for small datasets (1,360 images) and achieves accuracy comparable to human annotators for larger datasets (6,800 + images), enabling fully automated preliminary labeling. These findings demonstrate how domain-specific AI training can dramatically lower costs and accelerate database expansion while maintaining clinical relevance. This method not only benefits thyroid nodule analysis but also provides a scalable framework for other ultrasound-based diagnostic tasks, bridging the gap between AI development and real-world clinical workflows.

## 1. Introduction

The application of artificial intelligence (AI) in the medical field is booming, and AI-assisted medical image classification and recognition are gradually being deeply integrated into clinical practice, widely used in Computed Tomography (CT), Magnetic Resonance Imaging (MRI), pathological diagnosis, and ultrasound imaging-aided diagnosis [1–4], becoming a valuable assistant to doctors. AI models require targeted training to perform related tasks. AI model training methods are generally divided into unsupervised learning [5], semi-supervised learning [6], and supervised learning [7] based on whether supervision is present. For medical image classification tasks, the predicting results of supervised learning is closer to classification habits in clinical practice.

Supervised learning requires medical images to be annotated in advance, rely on meticulously and professionally annotation work. The quality of image data annotation directly affects model performance [8]. The annotation of ultrasound medical image data often requires preliminary annotation by junior physicians followed by review by senior physicians to ensure the accuracy of annotation information. Therefore, doctors’ experience directly determines the quality of image annotation, and experienced doctors are scarce and busy with clinical work, resulting in high costs for manual annotation, which limits the scale of image databases. The performance of AI is related to the size of the training data set; thus, how to reduce manual annotation costs and rapidly expand the database scale is a critical issue affecting AI model quality.

In medical imaging datasets, severe class imbalance poses a significant challenge to model training efficiency. Specifically, while common disease categories may account for over 30% of cases (e.g., benign thyroid nodules), rare pathological findings (such as medullary thyroid carcinoma) often constitute less than 0.1% of the total data set. This extreme distribution disparity forces models to prioritize majority class features during optimization, leading to compromised recognition capability for under-represented categories [9,10]. Studies have shown that data augmentation can effectively improve model training efficiency [11]. These augmentation methods are widely used for images captured by cameras, but the generation principles of ultrasound image data differ from those of camera-captured images. Therefore, how to specifically augment ultrasound images is an urgent problem to be solved to enhance training effectiveness with a fixed database scale.

Current research indicates that the accuracy of AI models in image annotation and classification can surpass manual annotation [12], making it a feasible solution to let AI models assist in annotation while learning.

This study takes thyroid nodule ultrasound images as an example to explore the feasibility of using AI to pre-annotate images to save manual annotation costs when dealing with small sample sizes and significant quantity differences between classes in ultrasound image data.

## 2. Experimental methods

### 2.1 Database image source and composition

This study selected 8,500 ultrasound images of various thyroid nodules from the workstation database of the Ultrasound Diagnostic Department of the 906th Hospital. These selected grayscale images do not contain color Doppler blood flow signals, indicator arrows, measurement marks, or other auxiliary marks. Each image contains at least one thyroid nodule. The sections include coronal (short-axis) and sagittal (long-axis) sections of the thyroid gland, as well as non-standard sections. We conducted privacy desensitization, ensuring that the images did not contain patient name information.

### 2.2 Database image annotation

We conducted two-stage annotation of ultrasound images. In the first stage, junior physicians (residents, physicians) annotated each image, and in the second stage, physicians above the deputy senior level (associate chief physicians, chief physicians) reviewed and corrected the annotations from the first stage.

We saved the annotation results from junior physicians in the first stage and the review results from senior physicians in the second stage. The model was trained using the annotated data set after the second-stage. We used the review results from senior physicians as the gold standard to calculate the misannotation rate of junior physicians in the first stage.

**Fig 1:**
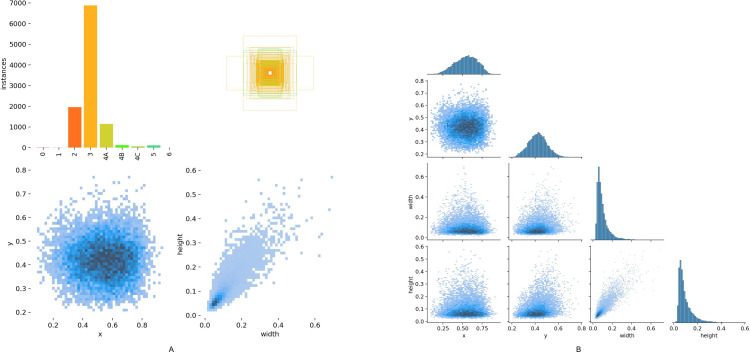
Distribution of nodule categories, sizes, and locations in the image data set. The bar chart in the upper left corner of Fig 1A represents the data quantities of each category (there are 6 thyroid nodule categories: 2, 3, 4A, 4B, 4C and 5) in the training set. Each colored bar represents the quantity of nodules in each category. In the upper right, lower left, and lower right corners of Fig 1A respectively show the sizes and quantities of bounding boxes for nodules, the positions of nodule centers relative to the entire image, and the aspect ratios of nodules relative to the entire image. The Fig 1B represents the relationships between the center coordinates x and y, and the width and height of the bounding boxes.

Various international standards for thyroid nodule classification exist, such as the 2017 ACR TI-RADS (Thyroid Imaging Reporting and Data System) by the American College of Radiology [13], the 2015 TI-RADS standard by the American Thyroid Association (ATA) [14], and the Korean TI-RADS [15]. In China, in addition to these international standards, the TI-RADS standard established by Shangyan Xu, Weiwei Zhan, et al., in 2016 [16], and the 2020 CTI-RAIDS classification [17] are also commonly used. Since our hospital consistently adopts the TI-RADS classification standard established by Shangyan Xu and Weiwei Zhan in 2016 [16], and the participating physicians are more familiar with this classification system, this study used this standard to annotate thyroid nodules in the images. This classification standard divides nodules into 2, 3, 4A, 4B, 4C, 5, and 6 categories. Since the 6 category in the classification standard is based on pathological diagnosis, not image morphology, only categories 2-5 are annotated in this study. For example, if an ultrasound image shows a nodule classified as 4B and the Fine Needle Aspiration Biopsy(FNAB) pathology result of this nodule indicates thyroid cancer, it should be annotated as category 6, but our database still annotates such images as category 4B based on their image manifestations. Fig 1 shows the quantity, size, and location distributions of these nodule categories.

### 2.3 Data augmentation

Since categories 4A, 4B, 4C, and 5 contain very few nodules, class imbalance may lead to poor training effectiveness [18]. Therefore, it is necessary to augment images containing these rare nodule types to improve AI model training effectiveness. In addition to conventional image augmentation methods such as brightness and contrast changes and small angle rotations [19], we specifically included ultrasound-specific augmentation methods such as defocus, acoustic shadow, and sidelobe artifacts. Studies have shown that using too many augmentation methods on a single image may not enhance training effectiveness [20], so we randomly applied only two augmentation methods to each image.

The ultrasound-specific augmentation methods adopted in this study are as follows

#### Simulated Defocus.

**Fig 2:**
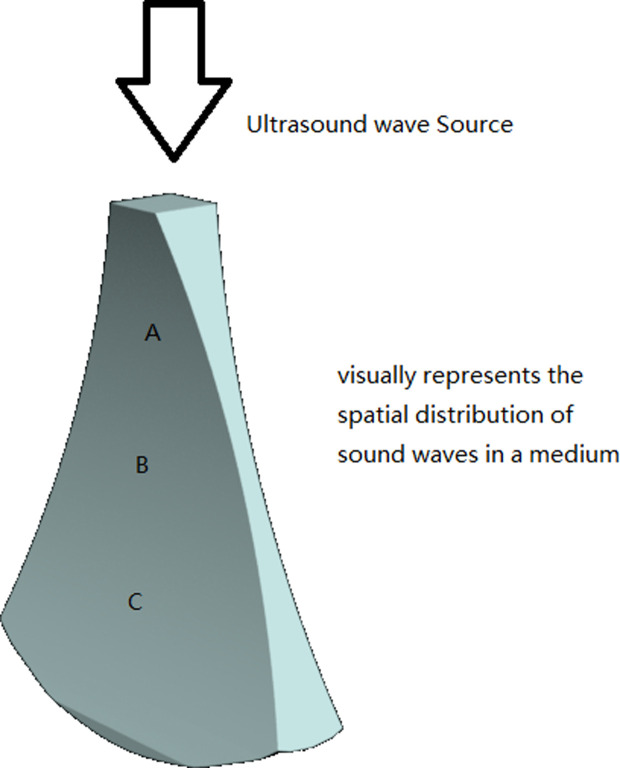
Visually represents the spatial distribution of sound waves in a medium. This diagram illustrates the sound field of ultrasonic waves emitted from an ultrasonic source (such as the transducer of an ultrasound probe) propagating through a medium. From the image, it can be observed that the ultrasonic waves propagate in a fan-shaped region within the medium, with the thinnest part of the fan-shaped region located at the center, which is the focal point of the sound. In the sound field, position B, where the fan-shaped region is thinnest, is the focal position, resulting in clearest imaging. In contrast, positions A and C, where the fan-shaped region is thicker, are in a defocused state, leading to blurred imaging.


Similar to optical imaging systems, ultrasound imaging systems may also experience out-of-focus situations, resulting in blurred images (Fig 2). We used Gaussian blur to blur the images to a certain extent, simulating defocus. Gaussian blur is a convolution operation where the Gaussian convolution kernel is a two-dimensional matrix whose element values are calculated based on the Gaussian distribution function. This convolution kernel is then convolved with each pixel and its neighboring pixels in the image to obtain new pixel values.

$$G(x,y)=\frac{1}{2\pi \sigma^{2}}\times e^{-\frac{x^{2}+y^{2}}{2\sigma^{2}}}\mathrm{\backslash ]}$$ (1)

Equation (1) is a two-dimensional Gaussian function. In this formula, x and y represent the horizontal and vertical distances of a pixel from the center point; σ is the standard deviation of the Gaussian distribution, controlling the intensity of the blur; $\frac{1}{2\pi \sigma^{2}}\;$ is the normalization coefficient to ensure the integral of the function equals 1.

**Fig 3:**
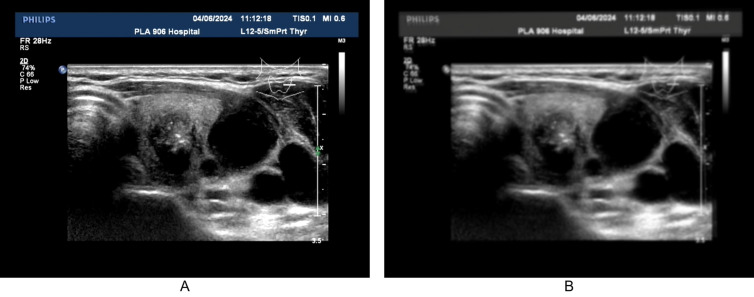
Comparison of images before and after defocus simulation. Fig 3 demonstrates the difference before and after defocus simulation, Fig 3A showing the original image and Fig 3B displaying the simulated defocused image.

We use Equation (1) to create a 5×5 convolution kernel and perform a 2D convolution on the image with a stride of 1. This functionality is implemented using the torch.nn.Conv2d function in PyTorch. Additionally, we randomize the σ value in Equation (1) to simulate the degree of image blurring (Fig 3).The program code implementing this functionality can be downloaded via the link provided at the end of the article, the function name is blur_image().

#### Simulated acoustic shadow.

Acoustic shadow is a common artifact in ultrasound imaging. Similar to how shadows are produced behind opaque objects in optical systems, acoustic shadows are dimmed areas produced when sound beams encounter calcified lesions or other tissues that are difficult for sound beams to penetrate. In this study, we wrote a code to randomly generate dimmed areas of random positions and sizes in the images.

**Fig 4:**
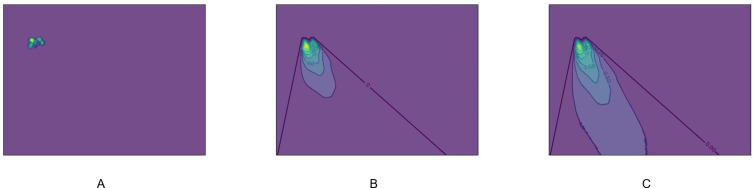
Demonstration of Acoustic Shadow Formation During Sound Beam Propagation. Fig 4 uses contour maps to demonstrate the concept of acoustic shadow formation behind a high-acoustic-impedance object during sound beam propagation. Fig 4A: Displays an irregularly shaped object with high acoustic impedance. Fig 4B: Shows the formation of an acoustic shadow behind the object as the sound beam propagates from the upper-left to the lower-right direction. Fig 4C: Illustrates how the acoustic shadow gradually weakens as the beam continues propagating further downward and rightward due to wave diffraction, with the shadow intensity decreasing as the distance from the object increases. In Fig 4B and Fig 4C brighter regions stand for stronger acoustic shadows.

**Fig 5:**
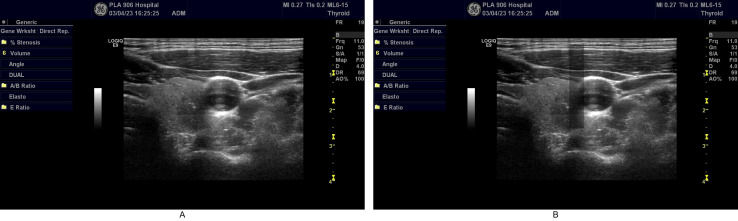
Simulating acoustic shadowing effects in ultrasound imaging. Fig 5A: A thyroid ultrasound image showing an ill-defined nodule within the thyroid gland. Fig 5B: A semi-transparent black square is partly overlaid on the nodule to simulate the acoustic shadowing effect, demonstrating how such artifacts can obscure nodule visibility.

We implemented a program to simulate the aforementioned acoustic shadow formation process, and Fig 4 was draw by the program. However, this method is computationally intensive and cannot be used in real-time applications. In this study, we instead employed randomly sized black boxes with adjustable transparency (alpha values) to approximate the acoustic shadow effect, specifically mimicking its masking effect on nodules (Fig 5).

#### Sidelobe artifacts.

**Fig 6:**
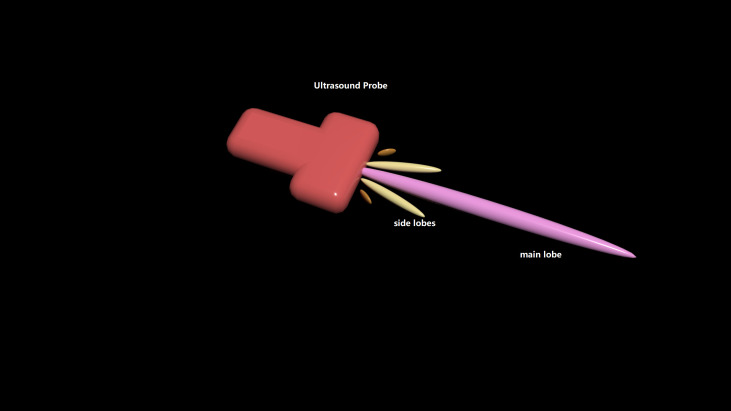
Schematic diagram illustrating the main lobe beam and side lobe beams emitted by an ultrasound transducer. Fig 6 is a schematic diagram of the ultrasound beam emitted by a transducer, illustrating the main lobe and side lobes of the acoustic beam. The ultrasound beam originates from the transducer on the left. The central, longest pink cone-shaped lobe represents the main lobe, which carries the majority of the acoustic energy. The four shorter cone-shaped lobes on either side represent side lobes, contributing only a small fraction of the total energy.

**Fig 7:**
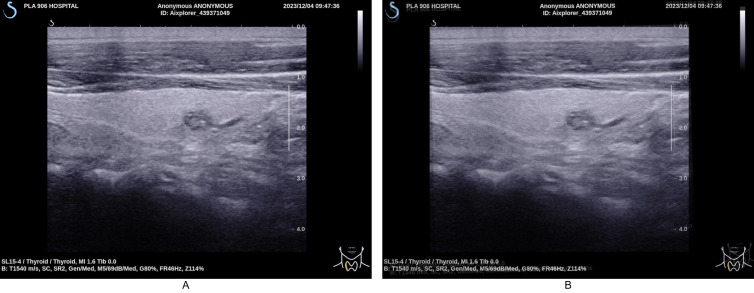
Sidelobe effect Simulated. Fig 7A is the original one, while Fig 7B is created by randomly rotating the original image by a small angle, then randomly adjusting its transparency (alpha value) and overlaying it onto the original image. This right image simulates the common sidelobe effect observed in ultrasound imaging.

**Fig 8:**
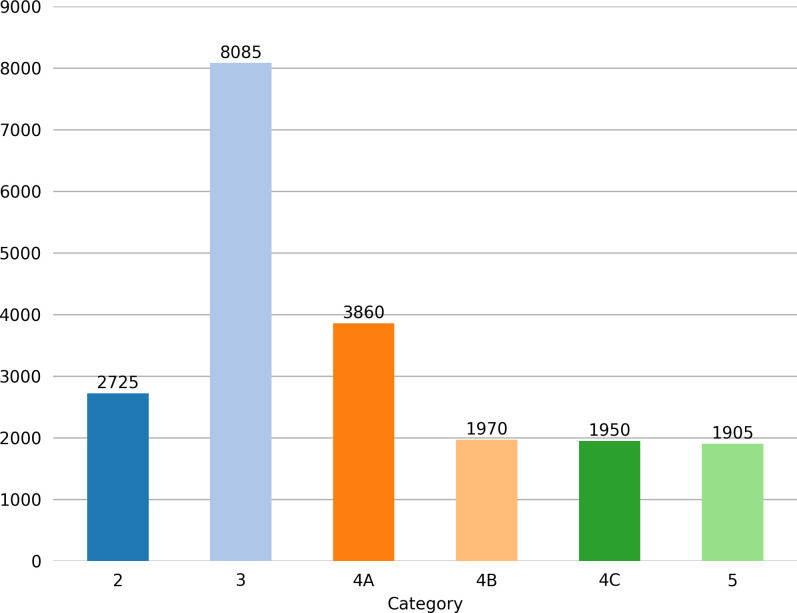
Bar chart of nodule category distributions after image augmentation. Fig 8 shows that although category 3 nodules still have the highest quantity, compared to Fig 1, the quantities of categories 4A, 4B, 4C and 5 have significantly increased. Since multiple nodules may appear in a single image, augmenting images with fewer nodules will also increase the quantity of category 3 nodules to some extent. Therefore, compared to Fig 1, the quantity of category 3 nodules has also increased slightly.

Due to the characteristics of ultrasound probes, side lobes interfere with the main beam (Fig 6), resulting in a relatively faint rotated and displaced original image superimposed on the image (Fig 7). We wrote a code to achieve this effect (Fig 8).

### 2.4 AI model selection

This study used the YOLOv8 model for training. YOLOv8 is a product of the YOLO architecture model family from Ultralytics [21], capable of object detection, instance segmentation, and image classification. This model, created in PyTorch [22], can run on both CPUs and GPUs. YOLOv8 is an accurate and flexible visual AI solution that excels in various visual technology tasks and demonstrates superiority in multiple aspects. YOLO means “You Only Look Once,” as it can annotate target types and coordinates throughout an image with a single scan. It is widely used in image classification, segmentation, and other fields.

### 2.5 AI model training

We trained the model in two stages: once without data augmentation and once with data augmentation. During the progressive training process, we employed an NVIDIA RTX 4090 GPU for computational acceleration. Training time expanded significantly with increasing data batches: the first batch (1,360 images) required approximately 30 minutes, while the final batch (6,800 + images) extended to 8 hours per session due to heightened model complexity and data volume. The entire iterative training workflow (consisting of 5 batches) achieved a total training time of ~16 hours, with efficiency optimizations realized through the GPU’s parallel computing capabilities (Tables 1 and 2).

#### 2.5.1 AI model parameter settings.

**Table 1:** Main parameters for AI model training.

No.	Parameter Name	Value	No.	Parameter Name	Value
1	Epochs	80	6	warmup_epochs	3
2	Image Size	800	7	warmup_momentum	0.8
3	batch size	40	8	lr0	0.01
4	momentum	0.937	9	lrf	0.01
5	weight_decay	0.0005	10	warmup_bias_lr	0.1
11	conf	0.1			

Table 1 legend: We primarily utilized the default settings of YOLOv8, with the exception of adjusting the confidence threshold (conf) to 0.1 to enhance the detection sensitivity for nodules. Additionally, the input image size was set to 800 to improve the model’s accuracy.

**Table 2:** Main parameters for image AI model prediction.

No.	Parameter Name	Value	No.	Parameter Name	Value
1	conf	0.1	3	iou	0.7
2	agnostic_nms	TRUE			

Table 2 legend: Both model training and image prediction utilized the NVIDIA RTX 4090 GPU for computational acceleration.

#### 2.5.2 Model training without data augmentation.

**Fig 9:**
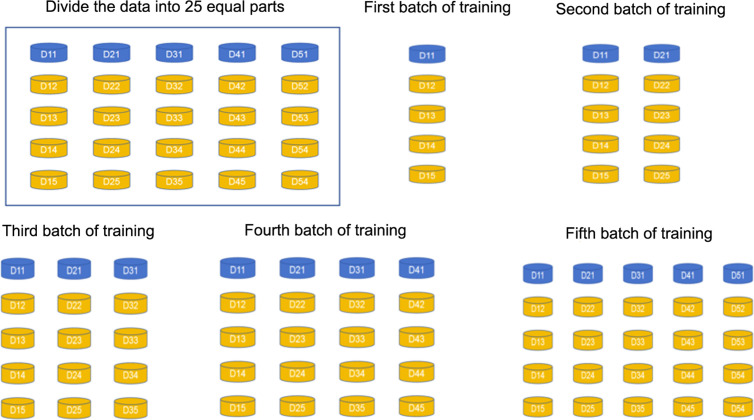
Data division for each batch of training. Fig 9 shows that the entire database was randomly divided into 25 equal portions and trained in 5 batches. The Nth batch used the first 5 × N portions of data as the database, with yellow portions serving as the training data set and blue portions as the validation data set. For example, in the second batch, we used the first 10 portions of data for training, with 8 yellow portions for the training data set and 2 blue portions for the validation data set.

We first trained the model without data augmentation to compare its effectiveness with training using augmented data. The data set was equally divided into 25 portions, and model training was conducted in 5 batches. The Nth batch used the first 5 × N portions of data in the database, with yellow portions serving as the training data set and blue portions as the validation data set (see Fig 9). For example, in the second batch, we used the first 5 × 2 = 10 portions of data for training, with 8 yellow portions for the training data set and 2 blue portions for the validation data set.

Each batch underwent 80 epochs of training. The network weight parameters of the first batch’s training model were randomly initialized, and after 80 epochs, the model with the best performance during training was saved. For the second to fifth batches, the network weight parameters of the best-performing model from the previous batch were used as the initial parameters. The model with the best performance during each batch was saved.

#### 2.5.3 Model training with data augmentation.

The data division and training method were the same as those for model training without data augmentation. First, the original database was randomly divided into 25 equal portions. Then, each small portion of data was analyzed to count the number of nodules in each category. And the images containing the categories that had fewer number were augmented to make the number of the categories increase.

The training method was consistent with that for model training without data augmentation, involving 5 batches of training with 80 epochs each. Except for the first batch, which used randomly initialized network weight parameters, the subsequent batches used the network weight parameters of the best-performing model from the previous batch.

## 3. Experimental results

### 3.1 Quality of junior physicians’ annotation of thyroid nodule categories in ultrasound images

**Fig 10:**
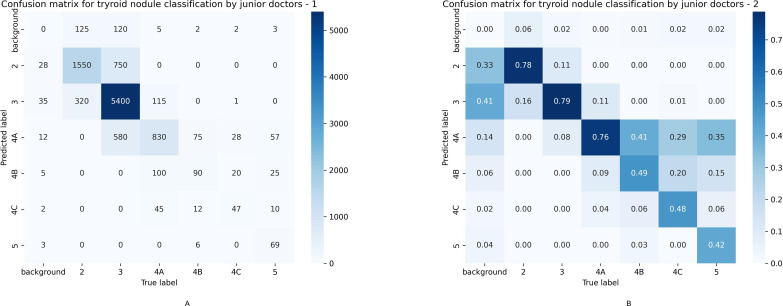
Confusion matrix of junior physicians’ annotation of thyroid nodule categories in ultrasound images. Fig 10 is a confusion matrix. Fig 10A is a proportional confusion matrix, and Fig 10B is a quantitative confusion matrix. It shows the accuracy of each nodule category and the misclassification categories. In the confusion matrix, the horizontal axis represents the correct classification after senior physician review, and the vertical axis represents the classification by junior physicians. It can be seen that the accuracy for categories 2, 3, and 4A nodules was close to 80%, while that for categories 4B, 4C, and 5 nodules was less than 50%. Categories 4B, 4C, and 5 nodules were most frequently misclassified as category 4A.

The overall accuracy of junior physicians’ annotation of thyroid nodule categories in ultrasound images was 76.9%. However, the accuracy varied greatly across categories. The accuracy for categories 2, 3, and 4A nodules was close to 80%, while that for categories 4B, 4C, and 5 nodules was less than 50%. Categories 4B, 4C, and 5 nodules were most frequently misclassified as category 4A. In a few images, junior physicians misclassified vascular, lymph node, and parathyroid structures within and around the thyroid gland as nodules (Fig 10).

Since categories 2, 3, and 4A nodules accounted for 95.7% of the total nodules, and categories 4B, 4C, and 5 nodules accounted for only 4.3%, the overall accuracy was 76.9%.

### 3.2 Model training results using the original data set

**Fig 11:**
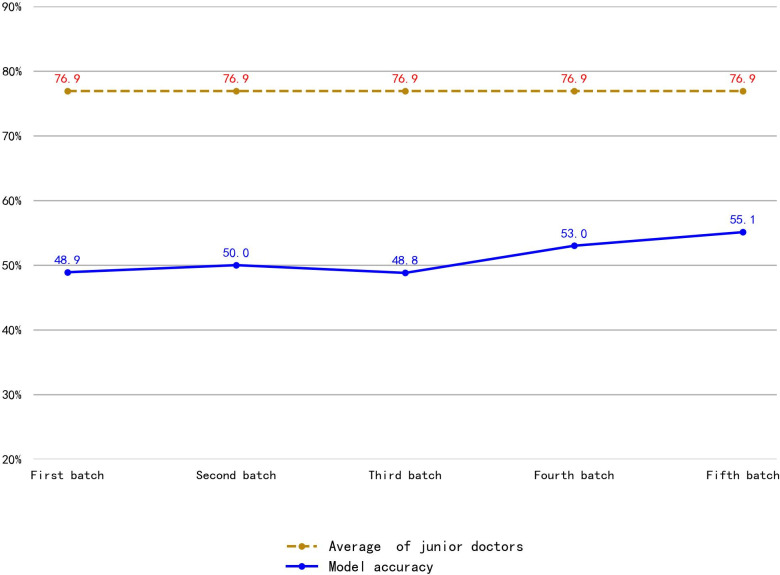
Comparison of nodule classification accuracy between junior physicians and the model trained using the original dataset. The orange dashed line in Fig 11 represents the average accuracy of junior physicians’ annotation of thyroid nodule categories in ultrasound images, and the blue line represents the average accuracy of the model trained using the original data set. It can be seen that junior physicians’ average accuracy was much higher than that of the model.

**Fig 12:**
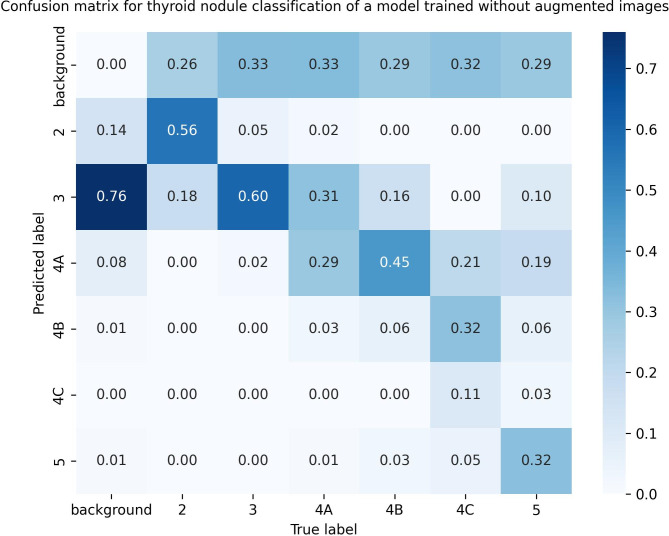
Confusion matrix of the model trained using the original data set. Fig 12 is a proportional confusion matrix of the model trained using the original data set for the fifth batch. It can be seen that the highest classification accuracy was 60% for category 3 nodules, and the lowest was 6% for category 4B nodules, both significantly lower than junior physicians’ accuracy shown in Fig 10.

The average accuracy of the model trained using the original dataset was 55.1% (Fig 11), significantly lower than that of junior physicians (Fig 12).

### 3.3 Model training results using the augmented data set

**Fig 13:**
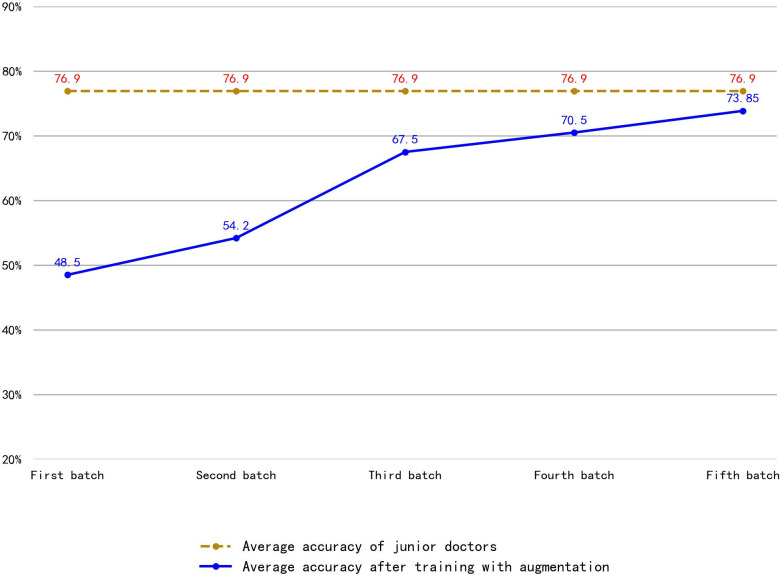
Comparison of Nodule Classification Accuracy in Ultrasound Images Between Junior Doctors and Models Trained with Augmented Data. The orange dashed line in Fig 13 represents the average accuracy of junior physicians’ annotation of thyroid nodule categories in ultrasound images, and the blue line represents the average accuracy of the model trained using the original data set. The average accuracy of nodule annotation in the augmented model has improved from 48.5% to 73.85%, very close to the accuracy level of junior doctors’ annotations.

Using the augmented data set, the first batch achieved an average accuracy of 48.5% for thyroid nodule classification in ultrasound images. The first batch’s training data set consisted of 1,360 original images and their augmented images, and the validation data set consisted of 340 original images and their augmented images. The fifth batch achieved an accuracy of 73.85%, very close to junior physicians’ accuracy of 76.9% (Fig 13).

**Fig 14:**
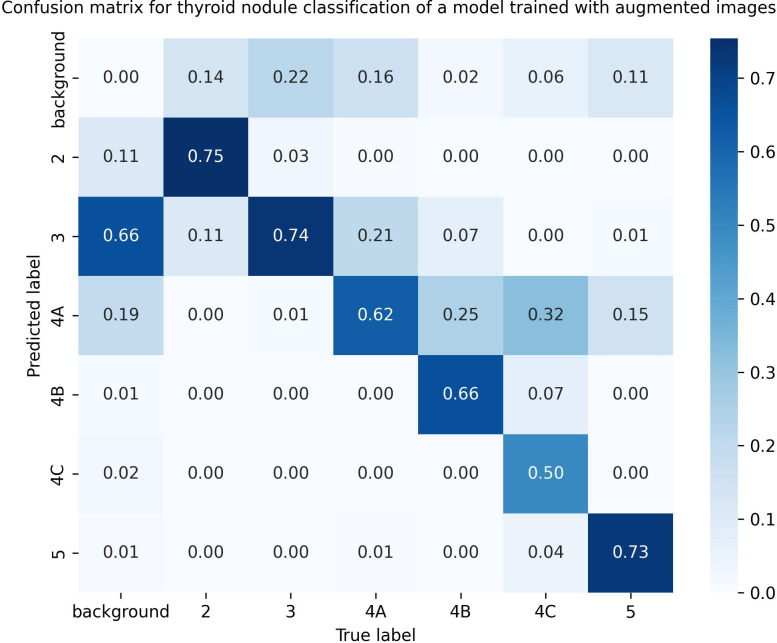
Confusion Matrix of Model Trained with Augmented Data for Thyroid Nodule Classification in Ultrasound Images. Fig 14 presents the confusion matrix for the classification of thyroid nodules in ultrasound images using the model trained with augmented data. It can be observed that the classification accuracy is lowest for category 4c nodules, at 50%, and highest for category 2 nodules, at 75%.

**Fig 15:**
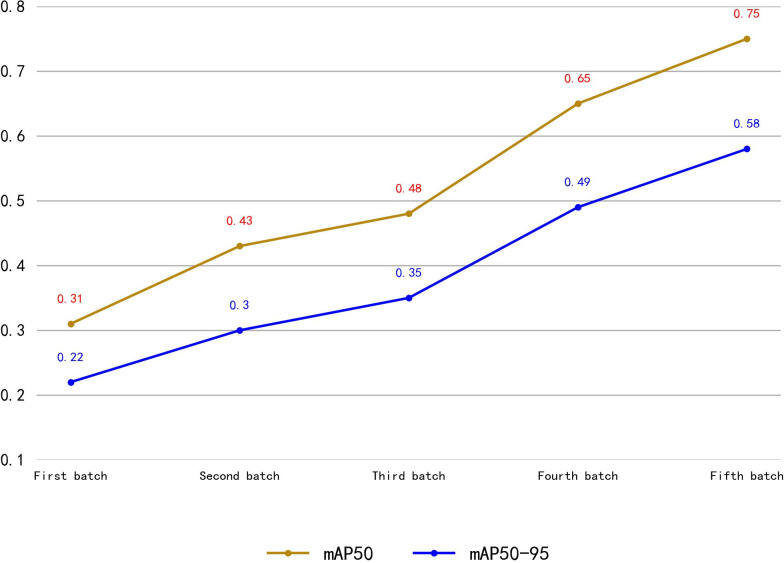
mAP50 and mAP50-95 of the Model Trained with Augmented Data. As shown in Fig 15, with the increase in training batches, both mAP50 and mAP50-95 continuously increase. After the fifth batch of training, the model’s mAP50 is 0.75, and mAP50-95 is 0.58.

By comparing Fig 8 and Fig 4, we found that junior physicians’ classification accuracy and that of the model trained on augmented data were very similar, but for categories 4A, 4B, 4C, and 5 nodules, the model’s accuracy was significantly higher than that of junior physicians. Junior physicians’ accuracy for these categories was less than 50%, while the model’s accuracy was around 70%, with the highest accuracy of 73% for category 5 nodules (Fig 14). For categories 2 and 3 nodules, the accuracy of both was very close (Fig 15). 

## 4. Discussion

This study demonstrates the feasibility of significantly reducing manual annotation costs in thyroid nodule ultrasound classification through a phased AI pre-annotation strategy combined with ultrasound-specific data augmentation techniques. These findings provide a novel technical pathway for efficient medical image database construction, particularly offering practical value for resource-limited small and medium-sized healthcare institutions.

Compared to traditional end-to-end annotation paradigms, the iterative pre-annotation mechanism proposed in this study exhibits unique advantages. When the original data set reached 6,800 images, the model achieved classification accuracy very close to junior physicians (76.9% vs. 73.85%) (Fig 13 ). The junior physicians annotation accuracy is aligning with findings from Christiansen et al. [23] in ovarian cancer ultrasound research and findings from Cheng et al. [24] in hepatocellular nodular lesions research. However, our phased training strategy reduced the required junior doctors’ annotation volume for model optimization by approximately 30% at the first round and 100% at the fifth round(as the AI model replaced the junior doctor to annotate the images). Notably, ultrasound-specific augmentation methods—including simulated defocus, acoustic shadows and sidelobe artifacts—effectively addressed the limitations of conventional geometric transformations in medical image enhancement.

Compared to the method used in Jingjiao’s universal ultrasound foundation model (USFM) research [25], our approach provides a novel framework for ultrasound image enhancement based on the physical mechanisms of ultrasound artifact generation. Additionally, in contrast to Generative Adversarial Networks (GANs) [26], our method achieves batch image generation with minimal computational resources. In this study, accelerated by an NVIDIA 4090 GPU, we generated over 10,000 images per minute. When compared to the Synthetic Minority Over-sampling Technique (SMOTE) [27], which synthesizes new samples by linearly interpolating neighboring minority-class instances in the feature space (potentially producing unrealistic synthetic images), our method generates images that better align with real-world ultrasound data in terms of anatomical plausibility and artifact patterns.

The reduction in annotation workload revealed in this study carries dual clinical significance: 1) It alleviates the review burden on physicians, allowing greater focus on complex case diagnoses; 2) It establishes a sustainable framework for continuous database expansion. Our approach demonstrates superior marginal cost reduction while maintaining annotation quality (validated through senior physician review). Remarkably, while junior physicians showed <50% accuracy in identifying 4B/4C/5-class nodules, the augmented model improved sensitivity for these rare categories up to 31% (the 5-class nodules), suggesting AI’s potential value in detecting high-risk nodules.

Despite the aforementioned strengths, this study has several limitations that warrant consideration. 1) Single-Center Data Source: The data were exclusively derived from a single hospital, limiting generalizability. Although images from 10 different type ultrasound machines across four brands were included to mitigate inter-device variability, this approach may not fully represent all commercially available systems. Future multicenter studies incorporating data from diverse institutions are planned to enhance reliability and external validity. 2) Static Image Analysis: The study focused solely on static 2D grayscale ultrasound images. While these provide high-resolution snapshots, dynamic video sequences—critical for real-world clinical lesion identification—were not evaluated. To address this gap, we intend to integrate video-derived frames into subsequent research to better simulate clinical workflows. 3) Limited Ultrasound Modalities: Only conventional 2D grayscale imaging was assessed, excluding advanced modalities such as shear wave elastography, contrast-enhanced ultrasound, and Doppler flow/power imaging. Whether our methodology applies to these techniques requires systematic validation in future investigations. 4) Generalizability of Augmentation Methods: The image augmentation strategies developed here may not extend to CT or MRI datasets due to fundamental differences in artifact generation mechanisms (e.g., beam-hardening artifacts in CT vs. susceptibility artifacts in MRI). Domain-specific adaptation will be necessary for cross-modality applications. 5)This study, using thyroid nodule classification in ultrasound images as an example, future research can explore similar tasks such as breast nodule classification, lymph node classification, and liver nodule classification in ultrasound images, Because they share the same imaging principles and artifact generation mechanisms.

## 5. Conclusion

This study validates the feasibility of a closed-loop “AI-assisted annotation → human verification → model iteration” system, offering novel insights to overcome medical AI’s data bottleneck. Ultrasound image tailored Image data augmentation can reduce the impact of poor model training performance caused by imbalance across ultrasound image categories. Through step-by-step annotation, training, and using the step-by-step trained models to assist in image annotation and bounding box selection, the efficiency of pre-annotation by junior doctors can be improved. Ultimately, this approach can replace the pre-annotation work of junior doctors, significantly reducing the cost of data construction.

## References

[1] Esposito G, Ernst B, Henket M, Winandy M, Chatterjee A, Van Eyndhoven S, et al. AI-based chest CT analysis for rapid COVID-19 diagnosis and prognosis: a practical tool to flag high-risk patients and lower healthcare costs. Diagnostics (Basel). 2022;12(7):1608. doi: 10.3390/diagnostics12071608 35885513 PMC9324628

[2] Hou Y, Jiang K-W, Wang L-L, Zhi R, Bao M-L, Li Q, et al. Biopsy-free AI-aided precision MRI assessment in prediction of prostate cancer biochemical recurrence. Br J Cancer. 2023;129(10):1625–33. doi: 10.1038/s41416-023-02441-5 37758837 PMC10646026

[3] Ba W, Wang S, Shang M, Zhang Z, Wu H, Yu C, et al. Assessment of deep learning assistance for the pathological diagnosis of gastric cancer. Mod Pathol. 2022;35(9):1262–8. doi: 10.1038/s41379-022-01073-z 35396459 PMC9424110

[4] Morshed A, Shihab AA, Jahin MA, Nahian MJA, Sarker MMH, Wadud MSI, et al. Ultrasound-based AI for COVID-19 detection: A comprehensive review of public and private lung ultrasound datasets and studies. 2024.

[5] Makris D, Ellis T, Black J. Bridging the gaps between cameras. In: Proceedings of the 2004 IEEE Computer Society Conference on Computer Vision and Pattern Recognition, 2004. CVPR 2004. 205–10. doi: 10.1109/cvpr.2004.1315165

[6] Bhunia AK, Chowdhury PN, Yang Y, Hospedales TM, Xiang T, Song YZ. Vectorization and rasterization: self-supervised learning for sketch and handwriting. 2021. doi: 10.48550/arXiv.2103.13716

[7] Tu E, Yang J. A review of semi supervised learning theories and recent advances. 2019. doi: 10.48550/arXiv.1905.11590

[8] He S, Kirovski D, Wu M. High-fidelity data embedding for image annotation. IEEE Trans Image Process. 2009;18(2):429–35. doi: 10.1109/TIP.2008.2008733 19116199

[9] Morris LGT, Tuttle RM, Davies L. Changing trends in the incidence of thyroid cancer in the United States. JAMA Otolaryngol Head Neck Surg. 2016;142(7):709–11. doi: 10.1001/jamaoto.2016.0230 27078686 PMC4956490

[10] Wiltshire JJ, Drake TM, Uttley L, Balasubramanian SP. Systematic review of trends in the incidence rates of thyroid cancer. Thyroid. 2016;26(11):1541–52. doi: 10.1089/thy.2016.0100 27571228

[11] Boudouh N, Mokhtari B, Foufou S. Enhancing deep learning image classification using data augmentation and genetic algorithm-based optimization. Int J Multimed Info Retr. 2024;13(3). doi: 10.1007/s13735-024-00345-5

[12] Liu YI, Kamaya A, Desser TS, Rubin DL. A bayesian network for differentiating benign from malignant thyroid nodules using sonographic and demographic features. AJR Am J Roentgenol. 2011;196(5):W598-605. doi: 10.2214/AJR.09.4037 21512051

[13] Tessler FN, Middleton WD, Grant EG, Hoang JK, Berland LL, Teefey SA, et al. ACR Thyroid Imaging, Reporting and Data System (TI-RADS): white paper of the ACR TI-RADS Committee. J Am Coll Radiol. 2017;14(5):587–95. doi: 10.1016/j.jacr.2017.01.046 28372962

[14] Haugen BR, Alexander EK, Bible KC, Doherty GM, Mandel SJ, Nikiforov YE, et al. 2015 American Thyroid Association Management guidelines for adult patients with thyroid nodules and differentiated thyroid cancer: the American Thyroid Association Guidelines task force on thyroid nodules and differentiated thyroid cancer. Thyroid. 2016;26(1):1–133. doi: 10.1089/thy.2015.0020 26462967 PMC4739132

[15] Hee SJ, Hwan BJ, Jin C, Joo HE, Ji-Hoon K, Hen LY, et al. Ultrasonography diagnosis and imaging-based management of thyroid nodules: revised korean society of thyroid radiology consensus statement and recommendations. Korean J Radiology. 2016;17(3):370–95. 10.3348/kjr.2016.17.3.370 PMC4842857 27134526

[16] Shangyan X, Weiwei Z, Jianqiao Z. Establishment of the imaging reporting and data system of thyroid micronodule. Chinese J Med Ultrasound (Electronic Edition). 2016.

[17] Zhou JQ, Yin LX, Wei X, Zhang S, Zhan WW. Chinese guidelines for ultrasound malignancy risk stratification of thyroid nodules: the C-TIRADS. Endocrine. 2020;70(9). doi: 10.3760/cma.j.cn131148-20210205-00092 32827126

[18] Eaton-Rosen Z, Bragman F et al.Improving data augmentation for medical image segmentation.In:MIDL(2018). Available from: https://openreview.net/forum?id=rkBBChjiG

[19] Frid-Adar M, Diamant I, Klang E, Amitai M, Goldberger J, Greenspan H. GAN-based synthetic medical image augmentation for increased CNN performance in liver lesion classification. Neurocomputing. 2018;321:321–31. doi: 10.1016/j.neucom.2018.09.013

[20] Lee LH, Gao Y, Noble JA. Principled ultrasound data augmentation for classification of standard planes. Lecture Notes in Computer Science. Springer International Publishing. 2021:729–41. doi: 10.1007/978-3-030-78191-0_56

[21] ultralytics.com. Ultralytics Inc.; 2025 [cited 2025 Jan 2]. Available from: https://www.ultralytics.com/

[22] pytorch.org. The PyTorch Foundation; 2025 [cited 2025 Jan 2]. Available from: https://pytorch.org/

[23] Christiansen F, Konuk E, Ganeshan AR, Welch R, Palés Huix J, Czekierdowski A, et al. International multicenter validation of AI-driven ultrasound detection of ovarian cancer. Nat Med. 2025;31(1):189–96. doi: 10.1038/s41591-024-03329-4 39747679 PMC11750711

[24] Cheng N, Ren Y, Zhou J, Zhang Y, Wang D, Zhang X, et al. Deep learning-based classification of hepatocellular nodular lesions on whole-slide histopathologic images. Gastroenterology. 2022;162(7):1948–1961.e7. doi: 10.1053/j.gastro.2022.02.025 35202643

[25] Jiao J, Zhou J, Li X, Xia M, Huang Y, Huang L, et al. USFM: A universal ultrasound foundation model generalized to tasks and organs towards label efficient image analysis. Med Image Anal. 2024;96:103202. doi: 10.1016/j.media.2024.103202 38788326

[26] Islam T, Hafiz MdS, Jim JR, Kabir MdM, Mridha MF. A systematic review of deep learning data augmentation in medical imaging: Recent advances and future research directions. Healthcare Analytics. 2024;5:100340. doi: 10.1016/j.health.2024.100340

[27] Nugroho HA, Frannita E legya, Hutami AHT, Chondah L, Nugroho A, Fauzi RN, et al. Deep Learning for Analyzing Thyroid Nodule Malignancy Based on the Composition Characteristic of the Ultrasonography Images. In: 2020 International Conference on Advanced Computer Science and Information Systems (ICACSIS), 2020. 77–82. doi: 10.1109/icacsis51025.2020.9263234

